# The Effect of Heat Stress and Vitamin and Micro-Mineral Supplementation on Some Mineral Digestibility and Electrolyte Balance of Pigs

**DOI:** 10.3390/ani12030386

**Published:** 2022-02-05

**Authors:** Arth David Sol Valmoria Ortega, László Babinszky, Xénia Erika Ozsváth, Ogonji Humphrey Oriedo, Csaba Szabó

**Affiliations:** 1 Department of Animal Nutrition and Physiology, Faculty of Agricultural and Food Sciences and Environmental Management, Institute of Animal Science Biotechnology and Nature, University of Debrecen, Böszörményi Street 138, 4032 Debrecen, Hungary; ortega.david@agr.unideb.hu (A.D.S.V.O.); babinszky@agr.unideb.hu (L.B.); 2 Doctoral School of Animal Science, University of Debrecen, Böszörményi Street 138, 4032 Debrecen, Hungary; 3 Department of Animal Science, Faculty of Agricultural and Food Sciences and Environmental Management, Institute of Animal Science Biotechnology and Nature, University of Debrecen, Böszörményi Street 138, 4032 Debrecen, Hungary; ozsvath.xenia@agr.unideb.hu; 4 Department of Agriculture, Livestock and Food Security, Veterinary Services Section, County Government of Makueni, Makueni 78-90300, Kenya; ogonjihumphrey18@gmail.com

**Keywords:** pig, heat stress, digestibility of minerals, electrolyte balance

## Abstract

**Simple Summary:**

The deleterious effects of heat stress and its induced stressors on health and intestinal integrity may compromise the performance of pigs. Their physiological and behavioral mechanisms to promote thermoregulation can potentially influence electrolyte losses and compromise digestive capacity. The varied response exhibited by pigs under different durations of exposure to high ambient temperature led to our study on pigs and (1) the effect of the duration of heat stress on the digestibility of some minerals, (2) the balance of major electrolytes, and (3) the alleviation capability of vitamins and micro-minerals supplemented in combinations and at higher levels than recommended for pigs. Our results revealed that heat stress alone did not affect the digestibility of the minerals studied. However, supplementation of high levels of vitamins and micro-minerals improved the digestibility of some minerals, including calcium, selenium, and zinc, despite the exposure of the pigs to heat stress. Heat stress caused a significant reduction in the plasma chloride concentrations of pigs, which indicated an imbalance. Vitamin and micro-mineral supplementation corrected this issue.

**Abstract:**

Heat stress (HS) can have detrimental effects on intestinal integrity and can jeopardize the digestibility performance in pigs. With prolonged exposure to heat, some thermoregulatory processes in pigs are potential causes for electrolyte imbalance. The adverse effects of HS on mineral digestibility and electrolyte balance are not widely studied and information on its abatement through vitamin and micro-mineral supplementation in combinations above the recommended level in pigs is limited. The aim of this study is to research this area. Thirty-six Danbred hybrid barrows (65.1 ± 2.81kg) were distributed among the four treatments (n = 9 per treatment): (1) thermo-neutral (19.5 ± 0.9 °C, RH- 85.9 ± 7.3%)+ control diet (TC) (NRC, 2012), (2) HS (28.9 ± 0.9 °C, RH- 60.4 ± 4.3%) + control diet (HC), (3) HS +diet with elevated levels of vitamins (vitamin E and C) and micro-minerals (Zn and Se) (HT1), and (4) HS + diet with further elevation of vitamins and micro-minerals (HT2). Plasma samples were collected on days 7 and 21 of the experiment to investigate electrolyte concentration. During the experimental period, feces samples were collected from pigs placed in digestibility cages (six pigs from each treatment) to investigate the digestibility of Ca, P, Na, Se, and Zn. HS did not decrease the digestibility of minerals, but elevated supplementation of the selected vitamins and trace minerals improved it significantly. HS caused a significant decrease of Cl^−^ (*p* < 0.01) in plasma, indicating an imbalance. In conclusion, pigs can have some resilience against heat stress in terms of mineral digestibility. Proper vitamin and trace mineral supplementation are key factors in the ability of pigs to overcome the negative effects of HS.

## 1. Introduction

Climate change is evident, and its impact on animal health, nutrition, and welfare is significantly deleterious [1]. The accompanying rise in temperatures causes heat stress (HS) to food animals, such as pigs, which dramatically suffer from its adverse effects [2,3]. Although many reports concur that HS has harmful effects, several research reports suggested that the vulnerability of pigs to the adverse effects of HS depends on the duration of exposure. In some cases, pigs tend to adapt and become less affected when acclimatized to such stressors over a longer period than over a shorter one [4,5]. This ability is most likely due to the biphasic pattern of the thermoregulatory response in pigs, which involves intense physiological strains at the onset of HS and causes adaptive changes that lower metabolic heat production and gradually improve performance with prolonged exposure [6].

Nevertheless, exposure to such stressors forces pigs to exhibit behavioral and physiological mechanisms (such as reducing feed intake, increasing water intake, and respiration rate) to reduce the metabolic heat load and maintain euthermia [7]. However, such means can impair the integrity of the gastrointestinal tract (GIT) and digestive function due to intestinal damage, hypoxia, and oxidative stress (OS), which in turn negatively influences the digestibility of nutrients in the heat-stressed animal [7,8,9,10]. HS can also promote electrolyte losses through excessive urination and evaporation, leading to electrolyte imbalance and jeopardizing the animal’s productive performance [7,8,9,10,11,12,13]. Moreover, as observed in pigs and broilers, HS can cause respiratory alkalosis and renal failure, exacerbating the situation [11,14].

Several dietary antioxidants, including vitamins (vitamin C and E) and micro-minerals (selenium and zinc), are known to mitigate some of the adverse effects of heat stress in pigs. The substances’ ability to avert cell damage and improve intestinal integrity and renal function can benefit animal performance under HS [15,16,17]. Supplementation of said vitamins and micro-minerals can also enhance the acid–base balance, as well as the metabolic and physiological functions of several species of food animals, which might influence the mineral digestibility and electrolyte balance of pigs suffering from HS [18,19].

Altogether, studies have demonstrated that the response to HS in pigs varies and the duration of exposure plays a role. Some dietary antioxidants are potential tools for the alleviation of HS adverse effects. However, there is limited information on whether different lengths of HS cause variable impacts on electrolyte balance and mineral digestibility, and whether elevated levels of vitamins (C and E) and micro-minerals (selenium and zinc), in combination, can improve said parameters. Therefore, the objective of this study is to examine whether the provision of elevated levels of these vitamins and micro-minerals would be beneficial to combat the adverse effects of HS.

## 2. Materials and Methods

All the experimental procedures were reviewed and approved by the University of Debrecen Animal Care Committee (Debrecen, Hungary—9/2019/DEMÁB).

### 2.1. Experimental Design, Animals and Diet

A total of thirty-six Danbred hybrid barrows weighing an average of 65.1 ± 2.81 kg were used in a trial conducted at the University of Debrecen, Institute for Agricultural Research and Educational Farm, Animal Husbandry Experimental Station (Kismacs, Debrecen, Hungary). The pigs were housed in groups of three on concrete floor pens (three pens per treatment, twelve pens in total), ad libitum access to feed and water was provided throughout the experiment. Before the experimental period, all pigs were allowed a seven-day adaptation period to their pens, fed ad libitum (with basal feed), and housed in a thermo-neutral environment (TN) (19.3 ± 1 °C, RH- 93 ± 2.9%). Afterward, the temperature of the thermo-neutral room, which housed nine pigs (three pens), was maintained at 19.5 ± 0.9 °C, RH- 85.9 ± 7.3% throughout the experiment. Meanwhile, the temperature of the HS room (nine pens altogether) was gradually raised to 30 °C for 7 days (heat increment period, day 1–7, HI) and the main period of the experiment commenced, which lasted 14 days (7 to 21 days of the trial).

The basal feed (C) was formulated on a corn–soybean meal basis according to the National Research Council (NRC, 2012) [20] recommendation for 75–100 kg live weight pigs having 155 g mean protein deposition per day (Table 1 and Table 2). Two additional dietary treatments (elevated 1 and elevated 2) were formulated by providing elevated levels of vitamins C and E and micro-minerals selenium and zinc as shown in Table 3. The pigs were distributed among four treatment groups, which consisted of a combination of environmental and dietary treatments: (1) thermo-neutral environment + basal diet (TC); (2) heat stress environment + basal diet (HC), (3) HS + diet with elevated levels of vitamins and micro-minerals (HT1), and (4) HS + diet with further elevation of vitamins and micro-minerals (HT2).

### 2.2. Digestibility Trial: Sample Collection and Analysis

In each treatment group, a total of six pigs (two per pen) were used for the digestibility trial. The assessment was performed in two periods (week 1 and week 2) right after the heat increment, with weekly changes to the experimental animal inside the digestibility cage derived from the various treatment replications. One period consisted of two days adaptation to the cage and five days of collection. Feces and feed residue were collected daily, pooled by cage, frozen at −20 °C, and sampled. Feed and feces samples were analyzed for dry matter (ISO 6496), crude ash (ISO 5984), calcium (Ca), phosphorus (P), sodium (Na), selenium (Se), and zinc (Zn), and an elementary analysis was carried out after 1–2g of samples were digested in a block digester (LABOR MIM, Budapest, Hungary) with 10 mL cc. Nitric acid at 60 °C for 30 min and 3 mL of 30% hydrogen peroxide (Sigma-Aldrich, Saint Louis, MI, USA) at 90 min at 120 °C. The digested samples were filled to 50 mL with distilled water and filtered through MN640W (155 mm) filter paper. The analysis was carried out with the ICP-OES technique (Optima 3300 DV, Perkin–Elmer, Waltham, MA, USA).

### 2.3. Blood Collection and Analysis

Blood samples were collected from the external jugular vein of the pigs on the first and last day of the experimental period (7th and 21st days of the trial) into EDTA tubes. The collected blood samples were then incubated at 4 °C for 20 min and then centrifuged at 3000 g for 15 min. The separated plasma samples were then stored at −80 °C, before analysis. Plasma concentrations of significant electrolytes (sodium (Na), potassium (K), and chloride(Cl^−^)) were used as markers for electrolyte balance with reference to previous studies [21,22,23,24], and as mentioned by Shrimanker and Bhattarai [25]. The analysis of the plasma samples was performed in triplicate. The plasma levels of Na, K, and Cl^−^ were analysed through the photometric method with a Lab-Analyse (Orvostechnika Ltd., Budapest, Hungary) half-automatic analyser. Distilled water (Na and K) and chloride reagent (Cl^−^) were used as a blank before measuring every sample in the Lab-Analyse kit.

### 2.4. Statistical Analysis

Data were analyzed with variance analyses using GraphPad Prism 8.4.3 software (Graph Pad Software Incorporated, San Diego, CA, USA). A two-way analysis of variance (ANOVA) was used to determine the effects of HS duration and vitamin and micro-mineral supplementation on mineral digestibility and electrolyte concentration. The data were expressed as a mean, with a means separation by Tukey’s multiple comparison test.

## 3. Results

### 3.1. Mineral Digestibility

There was a significant HS duration (period) effect observed in the case of sodium and zinc (*p <* 0.05) (Table 4). The environmental and dietary treatments significantly affected the fecal digestibility of all minerals (*p <* 0.05). As observed in both periods, pigs in the TC and HC groups had a similar mineral digestibility (*p >* 0.05), indicating that the genotype used in the trial has some resilience to HS. Elevated levels of some vitamins and trace minerals (treatment HT1) resulted in increased digestibility (*p >* 0.05) in the second week of the heat stress period compared to the TC treatment. Further elevation of vitamin C, vitamin E, Zn, and Se (treatment HT2) did not improve the digestibility of the minerals tested (*p <* 0.05).

### 3.2. Markers for Electrolyte Balance

Longer chronic heat stress elevates the plasma Na level (*p* < 0.05) (Table 5). The plasma levels of K were similar (*p* > 0.05) despite the thermal and dietary treatments. However, a significant reduction of plasma Cl^−^ (*p* < 0.05) was observed in pigs due to heat stress (HC group). The supplementation of vitamin and micro-minerals was fully or partly able to mitigate this negative effect.

## 4. Discussion

The pigs’ exposure to different durations of HS had varying responses with a high possibility of thermal acclimation at a more extended period of exposure [4,6]. HS is noted to have a deleterious impact on the intestinal integrity and digestive function of pigs, and it was reported that HS could negatively influence the nutrient and mineral retention capability [7,10,26]. Our results partly agree with Patience et al. [23] and Kim et al. [27], wherein no significant changes in the mineral digestibility of pigs reared under HS conditions were observed. Our results might be due to the pigs’ ability for thermoregulation, which provides an avenue for their acclimation to high ambient temperature [28]. As previously reported, pigs show improved tolerance to heat with the duration of exposure, resulting in a positive production performance [4,6]. Interestingly, a significant increase in Ca, P, Na, Se, and Zn digestibility was observed in pigs that were fed elevated levels of vitamins and micro-minerals (HT1 vs. TC) during week 2. Our findings agree with the results reported by Xie et al. [29]. The increased levels of Se and Zn in the HT1 and HT2 diets might influence their digestibility. The significant increase in Ca, P, and Na digestibility does not apply in this situation, as the contents of these minerals were similar in all diets. Such results might be attributed to vitamins and micro-minerals capability to improve the integrity of the animals’ gastrointestinal (GIT) tract through their antioxidant effect.

Vitamins E and C and micro-minerals Se and Zn are notable dietary antioxidants. Vitamin E works as a chain-breaking antioxidant that prevents the propagation of free radicals in membrane and plasma lipoproteins, while vitamin C has the capability of protecting cell membranes, DNA, cell proteins, and lipids against reactive oxygen species (ROS) during OS and is also essential in the regeneration of other antioxidants, such as alpha-tocopherol (vitamin E) and glutathione [30,31,32,33]. Se, which is absorbed in the duodenum and cecum by active transport through a sodium pump, acts as a dietary antioxidant by forming into selenoproteins and regulating endogenous enzymes’ activity. In contrast, Zn, which is absorbed by the small intestine by transcellular transport processes, activates antioxidant peptides and enzymes by inducing metallothionein expression, which is vital in protecting cells against ROS [34,35,36,37]. Several research reported that supplementation of these vitamins and micro-minerals improved the heat-stressed pigs’ intestinal integrity and function by enhancing the intestinal epithelial function, alleviating HS-induced OS and improving intestinal health [15,38,39,40]. Along with the impact of these substances on GIT’s integrity and functionality, they influence the utilization of other vitamins essential for absorbing minerals. Vitamin C and Zn are cofactors of vitamin D, which promotes the absorption of calcium and phosphorus (phosphate) in the intestine via active transport and diffusion. Phosphate is transported into the epithelial cell by cotransport with sodium (sodium phosphate cotransporter), which is enhanced by said vitamin [41,42,43,44,45].

Previous observations regarding the concentrations of significant electrolytes (Na, K, and Cl^−^) under HS suggested no changes, and, therefore, no losses of electrolytes were determined [23]. However, our results suggest otherwise—although such losses did not significantly affect all the studied parameters. Acute and chronic HS reduces metabolic rate, causes metabolism disorders, acute phase response, and respiratory alkalosis that might affect the electrolyte balance of pigs [11,46,47,48]. The concentration of plasma Cl^−^ obtained from pigs under HS + fed basal diet (HC) was significantly reduced (*p* < 0.01) and is below the reference range in pigs (94-106 mmol/l) [49], regardless of the duration of exposure, which is in contrast to the results observed by Pearce et al. [5] in pigs exposed to acute HS (2–6 h). Moreover, it was also reported that one to three-day HS exposure led to significant changes in plasma Cl^−^ concentration in pigs. However, after 7 days and 28 days, similar electrolyte concentrations were observed between pigs under HS and thermal comfort [50,51]. Such discrepancies might be due to the pigs’ blood pH changes. Progressive alkalinity of the pigs’ blood pH was reported in response to HS. Such a condition can cause metabolic alkalosis, one of the major causes of the reduction in Cl^−^ concentration of the blood [11,48,52]. Another possibility is the HS-induced hepatic cellular apoptosis, which can affect the regulation of blood Cl^−^ by the liver [53]. Supplementation of dietary antioxidants (vitamins and micro-minerals) has shown significant improvement on some of the blood biochemical parameters of pigs under various stressors [15,29,38,54]. In our study, the concentration of Cl^−^ was significantly improved (*p <* 0.01) in HT2 pigs after 7 days of exposure, and slight improvements were also observed after 21 days. This observation might be due to the capability of vitamins and minerals to improve the regulation of the pigs’ blood pH under HS and mitigate its adverse effect on the pigs’ liver. High-level vitamin E and selenium supplementation reportedly improved blood pH in pigs kept at high ambient temperatures [15,19]. Moreover, as observed by Liu et al. [55], supplementation of organic selenium beyond nutrient requirements (0.4 and 0.6 mg/kg in the diet) alleviated the adverse effects of chronic HS in pig liver.

## 5. Conclusions

Our study indicates that the deleterious effect of HS on the digestibility of minerals was not exhibited by the pigs used in the experiment. Supplementation of high vitamin and micro-mineral levels improved the digestibility of minerals (Ca, P, Na, Se, and Zn) in pigs, despite exposure to high ambient temperatures. HS causes a significant reduction in the plasma concentration of Cl^−^ in both short- and long-term chronic heat stress, indicating an imbalance.

## Figures and Tables

**Table 1:** Composition and calculated nutrient content of basal feed ^a^.

Ingredients	Inclusion Rate (%)	Nutrient	Calculated Value
Corn	78.68	Digestible energy, MJ/kg	14.24
Soybean meal	16.33	Crude protein, %	12.81
Plant oil	2.11	SID ^c^ Lys, %	0.78
Limestone	0.92	SID Met+Cys, %	0.45
MCP ^b^	0.80	SID Thr, %	0.49
L-Lys	0.30	SID Trp, %	0.14
DL-Met	0.01	Ca, %	0.59
L-Trp	0.03	Digestible P, %	0.23
L-Thr	0.06	Na, %	0.10
Salt	0.26		
Vit. and min. premix	0.50		

^a^ NRC (2012) recommendation for 75–100 kg live weight pigs having 155 g mean protein deposition per day; ^b^ mono-calcium phosphate; ^c^ standardized ileal digestible.

**Table 2:** Nutrient content of the pre-mixture used in the basal feed (in 1kg of pre-mixture) ^a^.

Nutrient	Unit	Amount
Zinc	mg/kg	9999
Cupper	mg/kg	1454
Iron	mg/kg	7281
Manganese	mg/kg	9999
Iodine	mg/kg	136
Selenium	mg/kg	32
Vitamin A	IU/kg	410,000
Vitamin D-3	IU/kg	82,000
Vitamin E	mg/kg	2205
Vitamin K-3	mg/kg	82
Vitamin B-1	mg/kg	62
Vitamin B-2	mg/kg	205
Ca-d-pantothenate	mg/kg	492
Vitamin B-6	mg/kg	164
Vitamin B-12	mg/kg	1
Biotin	mg/kg	5
Niacin	mg/kg	1026
Folate	mg/kg	25
Choline chloride	mg/kg	60,000

^a^ At or above NRC (2012).

**Table 3:** Dietary treatments (supplementation mg/kg).

Nutrient	Basal Feed ^a^	Elevated 1	Elevated 2
Vitamin C	0	150	300
Vitamin E	11	41	71
Zinc ^b^	50	100	150
Selenium ^b^	0.16	0.21	0.26

^a^ NRC (2012); ^b^ organic source.

**Table 4:** Effects of heat stress and vitamin and micro-mineral supplementation on the fecal digestibility (%) of some minerals in fattening pigs.

	Treatment					*p* Values	
Minerals	TC	HC	HT1	HT2	SEM	Period	Treatment
Calcium						0.3796	0.0115
Week 1	88.1	86.2	91.4	89.0	0.71		
Week 2	86.0 ^b^	90.1 ^ab^	91.4 ^a^	90.1 ^ab^	0.85		
Phosphorus						0.1103	0.0113
Week 1	90.1 ^ab^	86.9 ^b^	91.9 ^a^	90.4 ^ab^	0.70		
Week 2	87.5 ^b^	91.7 ^ab^	92.6 ^a^	92.5 ^a^	0.79		
Sodium						0.0004	0.0012
Week 1	92.0 ^ab^	90.3 ^b^	94.5 ^a^	94.0 ^a^	0.57		
Week 2	87.4 ^b^	89.4 ^ab^	91.3 ^a^	91.9 ^a^	0.69		
Selenium						0.0720	<0.0001
Week 1	67.8 ^b^	61.4 ^b^	82.4 ^a^	86.3 ^a^	3.24		
Week 2	55.3 ^c^	66.1 ^bc^	77.2 ^ab^	83.6 ^a^	3.56		
Zinc						0.0166	<0.0001
Week 1	78.1 ^b^	70.1 ^b^	90.0 ^a^	89.8 ^a^	2.82		
Week 2	63.7 ^b^	72.1 ^b^	85.9 ^a^	86.1 ^a^	3.05		

^a,b,c^ means in a row with the same superscripts do not differ (*p* > 0.05); TC- thermo-neutral, fed control diet; HC- heat stress, fed control diet; HT1- heat stress, fed diet containing elevated levels of vitamins (C and E) and micro-minerals (Se and Zn); HT2- heat stress, vitamin, and micro-mineral increase doubled.

**Table 5:** Effects of heat stress and vitamin and micro-mineral supplementation on the plasma concentration (mmol/l) of major electrolytes as markers of electrolyte balance in pigs.

	Treatment					*p* Values	
Electrolytes	TC	HC	HT1	HT2	SEM	Time	Treatment
Sodium						0.0315	0.2798
day 7	204.3	194.0	205.7	213.1	2.95		
day 21	219.7	210.7	210.8	213.6	3.12		
Potassium						0.1540	0.3365
day 7	8.9	7.2	7.9	8.9	0.36		
day 21	9.6	9.0	8.7	8.5	0.34		
Chloride						0.2098	0.0013
day 7	100.3 ^a^	88.5 ^b^	96.2 ^ab^	100.9 ^a^	1.60		
day 21	104.7 ^a^	93.3 ^b^	97.7 ^ab^	101.0 ^ab^	1.65		

^a,b^ means in a row with the same superscripts do not differ *p* > 0.05.TC- thermo-neutral, fed control diet; HC- heat stress, fed control diet; HT1- heat stress, fed diet containing elevated vitamins (C and E) and micro-minerals (Se and Zn); HT2- heat stress, vitamin and micro-mineral increase doubled.

## Data Availability

The data presented in this study are available on request from the corresponding author.
